# A single ultrasensitive assay for detection and discrimination of tau aggregates of Alzheimer and Pick diseases

**DOI:** 10.1186/s40478-020-0887-z

**Published:** 2020-02-22

**Authors:** Michael A. Metrick, Natália do Carmo Ferreira, Eri Saijo, Allison Kraus, Kathy Newell, Gianluigi Zanusso, Michele Vendruscolo, Bernardino Ghetti, Byron Caughey

**Affiliations:** 1 LPVD, Rocky Mountain Laboratories, NIAID, NIH, Hamilton, MT 59840 USA; 2 Centre for Misfolding Diseases, Department of Chemistry, University of Cambridge, Cambridge, CB2 1EW UK; 3 Department of Pathology and Laboratory Medicine, Indiana University School of Medicine, Indianapolis, IN 46202 USA; 4 Department of Neurosciences, University of Verona, 37129 Verona, Italy

**Keywords:** RT-QuIC, Tau, Pick disease, Alzheimer disease, Chronic traumatic encephalopathy, Neurodegeneration, Protein misfolding, Seed, Primary age-related tauopathy

## Abstract

Multiple neurodegenerative diseases are characterized by aggregation of tau molecules. Adult humans express six isoforms of tau that contain either 3 or 4 microtubule binding repeats (3R or 4R tau). Different diseases involve preferential aggregation of 3R (e.g Pick disease), 4R (e.g. progressive supranuclear palsy), or both 3R and 4R tau molecules [e.g. Alzheimer disease and chronic traumatic encephalopathy]. Three ultrasensitive cell-free seed amplification assays [called tau real-time quaking induced conversion (tau RT-QuIC) assays] have been developed that preferentially detect 3R, 4R, or 3R/4R tau aggregates in biospecimens. In these reactions, low-fg amounts of a given self-propagating protein aggregate (the seed) are incubated with a vast excess of recombinant tau monomers (the substrate) in multi-well plates. Over time, the seeds incorporate the substrate to grow into amyloids that can then be detected using thioflavin T fluorescence. Here we describe a tau RT-QuIC assay (K12 RT-QuIC) that, using a C-terminally extended recombinant 3R tau substrate (K12CFh), enables sensitive detection of Pick disease, Alzheimer disease, and chronic traumatic encephalopathy seeds in brain homogenates. The discrimination of Pick disease from Alzheimer disease and chronic traumatic encephalopathy cases is then achieved through the quantitative differences in K12 RT-QuIC assay thioflavin T responses, which correlate with structural properties of the reaction products. In particular, Fourier transform infrared spectroscopy analysis of the respective K12CFh amyloids showed distinct β-sheet conformations, suggesting at least partial propagation of the original seed conformations in vitro. Thus, K12 RT-QuIC provides a single assay for ultrasensitive detection and discrimination of tau aggregates comprised mainly of 3R, or both 3R and 4R, tau isoforms.

## Introduction

Recent calls for better biomarkers of neurodegenerative diseases [20] have elicited efforts to detect disease-associated protein aggregates in diagnostically-accessible tissues. Among the novel approaches employed are those exploiting the self-propagating property of amyloid or pre-amyloid aggregates, that is, the ability to act as seeds that can grow by incorporating more monomers of their constituent proteins by seeded polymerization. Seeding activities in biospecimens from disease cases can be amplified by many orders of magnitude in vitro by using recombinant protein monomers as substrates and either sonication [1] or shaking [3, 9] to accelerate polymerization. The formation of amplified amyloid products in such reactions can be detected over time in multi-well plates using the amyloid-sensitive dye thioflavin T (ThT) [9, 33]. For example, real time quaking-induced conversion (RT-QuIC) assays have been adapted to the ultrasensitive detection of prions [2, 18, 24–26, 32, 33] as well as pathologic forms of α-synuclein [4, 8, 13, 19, 30] and tau [21, 23, 28]. These assays are providing a basis for accurate molecular diagnosis of the associated neurodegenerative diseases [5, 18, 35].

**Fig. 1:**
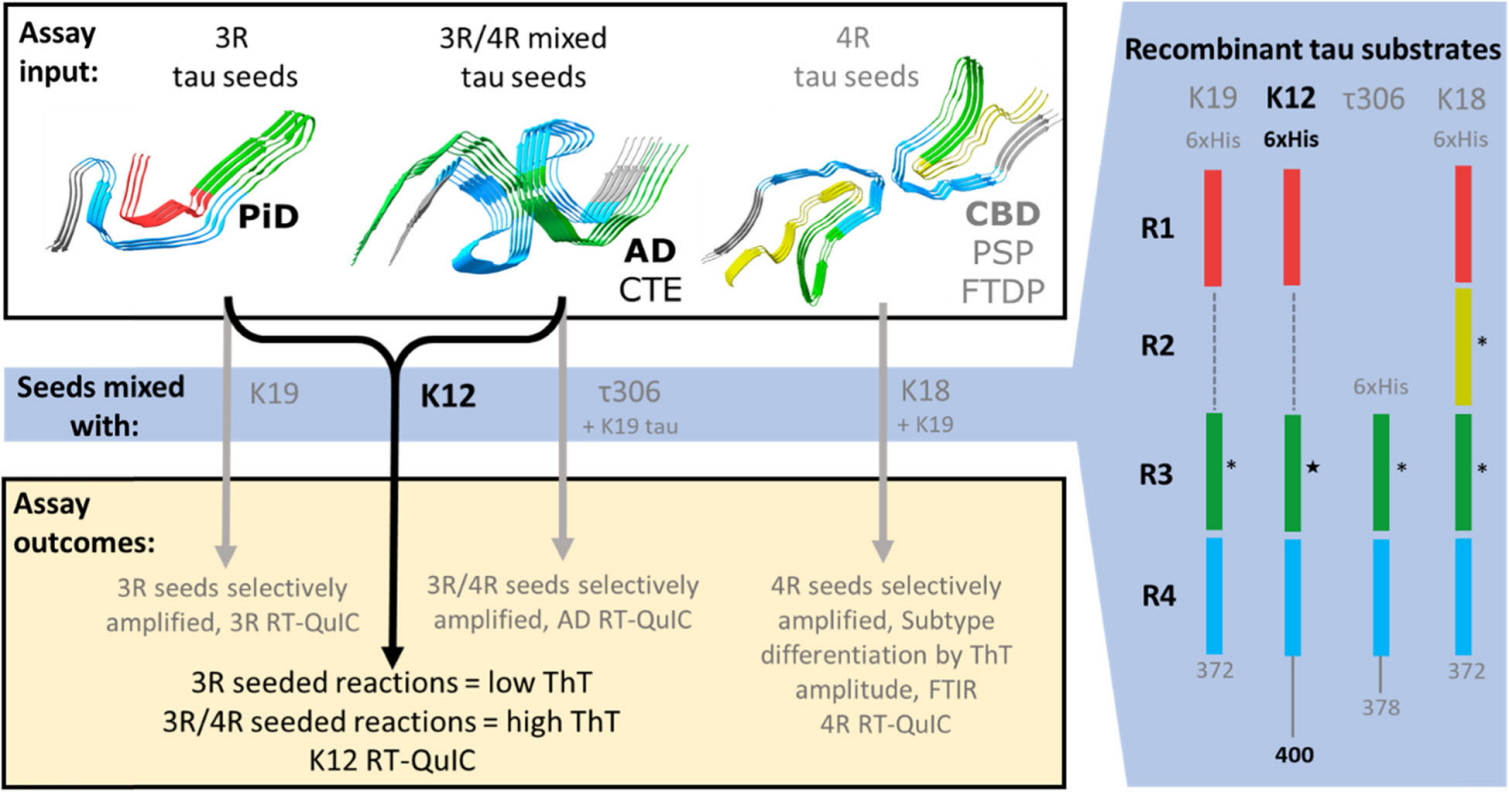
Comparison of the K12 tau RT-QuIC assay described in this work with the other three currently available tau RT-QuIC assays. Fibril structures were taken from PDB files originally published in [14–16, 36]. Cysteines in the canonical tau sequences were mutated to serines (*) or alanine (★) for recombinant tau fragments used in tau RT-QuIC assays. The 3R, AD, and 4R RT-QuIC assays mentioned in this figure have been described previously [21, 23, 27, 29]

Diseases involving tau pathology include those with preferential aggregation of 3 microtubule binding-repeat (3R), 4-repeat (4R), or both 3R and 4R (3R/4R) tau isoforms. Previously, our lab has developed tau RT-QuIC assays optimized to detect the mainly 3R aggregates of Pick disease (PiD) [27]; the 3R/4R aggregates of Alzheimer disease (AD) and chronic traumatic encephalopathy (CTE) [21]; or the 4R aggregates of progressive supranuclear palsy (PSP), corticobasal degeneration (CBD), frontotemporal dementias associated with *MAPT* mutations (FTDP 17 *MAPT*) and others [29]. In these assays, tau seeds from crude brain homogenates or cerebrospinal fluid (CSF) are added to reaction mixtures containing purified tau fragments truncated at different points in the microtubule binding domains to help confer seeding selectivity. For instance, the modified 3R tau fragment K19CFh forms fibrils when seeded with PiD brain homogenate as dilute as 10^− 9^, but not with PSP brain homogenate even at a 10^5^-fold higher concentration [27]. In contrast, the modified 4R tau fragment K18CFh amplifies 4R tau aggregates [29], and a tau fragment spanning the cross-β core of AD tau fibrils (τ306–378) selectively amplifies mixed 3R/4R tau seeds [21]. Figure 1 schematically represents the recombinant tau fragments used in different tau RT-QuIC assays.

Recently, structures of tau filaments of AD, CTE, and PiD have been solved by cryo-electron microscopy [14–16, 36]. These structures reveal that the 3R tau filaments characteristic of PiD differ in conformation from the 3R/4R filaments of AD and CTE. Since we previously achieved selective amplification of 3R PiD seeds with K19CFh, and amplification of 3R/4R AD and CTE seeds with τ306–378, we investigated whether a hybrid tau fragment could be seeded by PiD, AD, and CTE tau aggregates. We chose an extended 3R substrate that included amyloid core residues of both the PiD and AD tau filaments. This tau fragment, henceforth referred to as K12CFh (K12 cysteine-free and histidine tagged), spans the first, third and fourth repeats (R1, R3 and R4, respectively), extends to residue 400, and is a modification of the previously described K12 construct [34]. Our new tau assay, K12 RT-QuIC, detects 3R and 3R/4R tau seeds with similar sensitivity while at the same time discriminating PiD seeds from the others based on conformational differences between the amplified RT-QuIC products.

## Methods

### Brain tissue samples and compliance with ethical standards

Deidentified post-mortem brain samples were obtained from sources indicated in Table 1 and Acknowledgements in [21]. As samples were obtained from deceased, de-identified, consenting individuals, no further ethical approval was required.

**Table 1:**
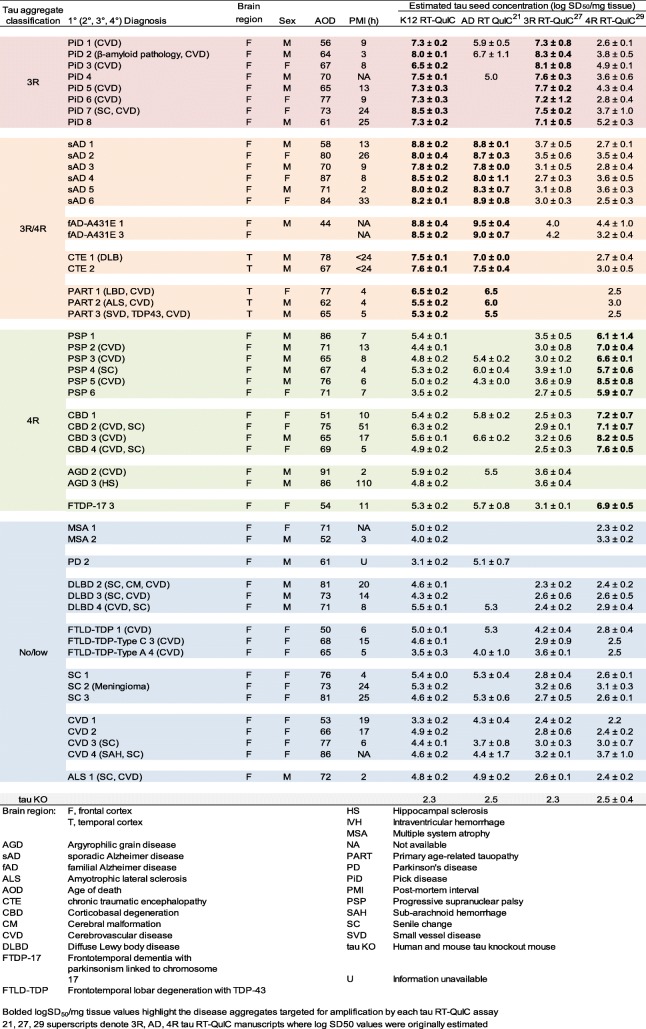
End-point quantification of tau seeding activity in brain tissue across tau RT-QuIC assays

### Neuropathology

Brains analyzed in this work were prepared as previously described with the numbering of patients following that in Table 1 in [21]. Briefly, one half of a patient brain was formalin fixed and the other half frozen. Diagnoses of fixed tissues were made using the stains described in [6, 21]. Tissue samples in this study were collected from frontal cortex except for CTE and primary age-related tauopathy (PART) samples which were from temporal cortex. 10% w/v brain homogenates of frontal cortex tissue were prepared in ice-cold PBS using 1 mm silica beads (BioSpec, 11079110z) and Beadbeater (BioSpec) or BeadMill 24 (Fischer). K. N. provided CTE; B. G. provided all other samples, PiD, AD, diseases with 4R tau deposits, and controls lacking tau pathology.

### Purification of recombinant K12CFh tau

A cloning cassette encoding the K12 tau fragment (residues 244–275 and 306–400 of the full-length human tau sequence) with an alanine mutation at residue 322 [17], thereby rendering it cysteine-free (K12CF), was introduced between the *Ndel* and *XhoI* sites of the pET28 bacterial vector resulting in a histidine tag at the N-terminal (K12CFh). The cysteine was mutated in order to prevent disulfide-mediated dimerization of the K12 molecules. Purification of K12CFh follows the steps described in [27, 28] and is summarized schematically in Additional file 1. Briefly, K12CFh was expressed in BL21(DE3) *E. coli* following the overnight autoinduction protocol described in [27]. Cells were centrifuged at 3750 rpm for 35 min at 4 °C, resuspended in buffer A (10 mM Tris, 500 mM NaCl, 5 mM imidazole, pH 8.0), and probe sonicated 3 × 45 s at 25% pulse with 15 s pause. Lysates were filtered through 0.45 μM syringe filter prior to nickel affinity chromatography using a His-Trap FF (GE Healthcare 17–5255-01) column. Sequential wash steps with 13 and 21% buffer B (10 mM Tris, 500 mM NaCl, 200 mM imidazole, pH 8.0) over 5 and 7 column volumes each eluted two contaminants observed previously in K18CFh, K19CFh, and τ306–378CFh purifications. K12CFh was eluted over a linear gradient of 46–200 mM imidazole over 8 column volumes. Fractions were pooled and precipitated in 4 x volumes of ice-cold acetone in 4 °C overnight. Acetone precipitant was pelleted at 10,000 x *g* for 20 min and washed with a further 20 mL acetone with a repeat centrifugation at 10,000 x *g* for 20 min. Acetone was decanted and pellets dissolved in 8 M Guanidine-HCl (GdnHCl) in PBS. The dissolved K12CFh was desalted into PBS, pH 7.0 using PD-10 desalting columns (GE Healthcare, 17–0851-01). Removal of guanidine was verified via UV absorbance readings. The K12CFh concentration was adjusted to ~ 0.75 mg/mL and aliquots stored at − 70 °C until use.

### K12 RT-QuIC

The K12 RT-QuIC assay follows a modified version of the protocols described in [27, 28]. K12CFh was thawed from − 70 °C and filtered through 100 kDa filters (Pall) to remove preformed aggregates. Concentration was adjusted to 6.5 μM ~ 0.1 mg/mL K12CFh in a buffer containing 40 mM HEPES, pH 7.4, 400 mM NaF, 40 μM heparin, and 10 μM thioflavin T (ThT). These reaction conditions were reached following the framework described in [23]. The K12CFh solution was thoroughly mixed in a polypropylene boat by gentle rocking for ~ 10 s and 48 μL or 49 μL mix was added to each well of a 384-well optically clear bottom plate (NUNC) using a multichannel pipettor. Brain homogenates (BHs) were thawed from storage at 10% w/v in ice cold PBS and serially diluted in 10-fold steps using a dilution buffer containing 0.53% tau-free mouse (KO; B6.129S4(Cg)-Mapt^tm1(EGFP)Klt^/J from Jackson Laboratories) and 1x N-2 Supplement (Gibco) + 10 mM HEPES. Inclusion of tau-free mouse BH and N-2 supplement were critical for preventing spontaneous aggregation of the K12CFh tau fragment. One or two μL of BH dilutions were seeded into quadruplicate or octuplicate wells for a final reaction volume of 50 μL in each well. Plates were sealed with clear adhesive sealing tape and placed in an Omega FLUOStar plate reader pre-warmed to 42 °C and subjected to rounds of 1 min shaking, 500 rpm, orbital, and 1 min rest, with ThT fluorescence reads (450 excitation, 480 emission) taken every 15 min.

### FTIR analysis of K12 RT-QuIC products

Once ThT fluorescence reached a plateau, and prior to spontaneous fibril formation in mock-seeded control (tau KO-seeded) wells, reactions were stopped and RT-QuIC products were recovered by scraping the bottom of the microplate wells with a pipette tip. Eight replicate reactions seeded with a 10^− 4^ dilution of the designated BH were pooled in 0.5 mL microfuge tubes and centrifuged at 13,100 x *g* for 10 min, at 4 °C. Supernatant containing soluble K12CFh and other buffer components was discarded, and pellets were washed twice with 400 μL H_2_O. Pellets were resuspended in 5 μL H_2_O and subjected to attenuated total reflectance (ATR) Fourier transform infrared (FTIR) spectroscopy. 1.5 μL H_2_O-protein slurry was applied to a Perkin-Elmer Spectrum100 FTIR with an ATR diamond attachment and dried with a gentle flow of dry air. Prior to scanning, sample and electronics chambers were purged with a constant flow of dry air. 100 replicate scans were averaged from 4000 to 800 cm^− 1^, normalized to amide I intensity (~ 1630 cm^− 1^ peak), and second derivatives were taken with 9 points for slope analysis.

### Electron microscopy of K12 RT-QuIC products

Prior to pelleting RT-QuIC products for FTIR, ~ 50 μL of pooled reactions seeded with 10^− 4^ BH dilutions were taken and wicked onto grids for transmission electron microscopy (TEM). Ultrathin C film on lacey carbon support film (400 mesh, Copper, TedPella Prod# 01824) were glow discharged and incubated with fibril solutions for 30 min at room temperature. Grids were washed 3 times with Milli-Q (Millipore)-purified water prior to being stained with Nano-W (Nanoprobes #2018–5) stain for 15 min and wicked dry. Images were acquired on a T12 (Thermo Fisher) transmission electron microscope operating at 120 kV with a Rio (Gatan) CMOS camera.

### LogSD_50_ and standard error statistical calculation

As a measure of seed concentrations in samples, we employed logSD_50_ calculations using the Spearman-Kärber method, which determine the log_10_ dilution at which 50% of wells give ThT fluorescence greater than 100 times the standard deviation of baseline fluorescence (threshold), prior to 60 h [11]. LogSD_50_/mg tissue values represent a determination from a single serial dilution of a brain homogenate with eight replicate reactions at dilutions from 10^− 3^ –10^− 10^, with a final adjustment to report SD_50_ seeding units per mg brain tissue. LogSD_50_* = x*_*p*  =  1_ + 1/2*d* - *d*∑*p* with *x*_*p*   =  1_ being the highest log dilution giving 8/8 positive responses; *d*   =   log dilution factor; *p*  =   proportion positive at a given dose; ∑*p*  =   the sum of values of *p* for *x*_*p*   =  1_ and all higher dilutions [11]. Error bars represent the standard error calculated with the formula [∑(*p*(1-*p*))/n-1)]^1/2^ where n = number of replicates. This method of error estimation is further described in [11].

### Analytical sensitivity estimation

To estimate the analytical sensitivity of K12 tau RT-QuIC, synthetic K12CFh fibrils were assayed over serial dilutions of a known concentration of starting fibrils. Fibrils were generated by seeding 6.5 μM K12CFh substrate with either AD, PiD, or KO BH at 10^− 4^ dilution in conditions identical to those described above for K12 RT-QuIC. Fibrils were scraped from the microwell plate and analyzed by SDS-PAGE for percent conversion of substrate to approximate the concentration of fibrils generated. We estimated that nearly ~ 100% of the K12CFh was converted in all reaction conditions. Serial dilutions of fibrils was conducted identically to those for brain homogenate, in a background of 10 mM HEPES pH 7.4, 1x N-2, and 0.53% KO BH.

## Results

### K12CFh fibrillizes in the presence of 3R and 3R/4R tau seeds

**Fig. 2:**
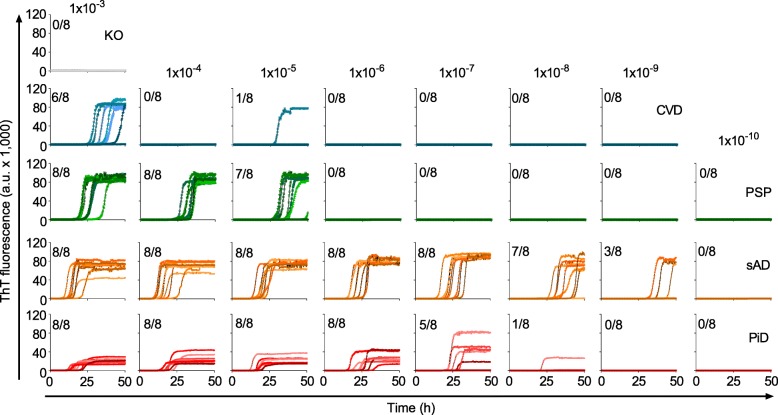
Primary ThT fluorescence data of K12 RT-QuIC endpoint dilution analyses. Panels show traces from 8 replicate reactions at the designated dilutions of tau-free mouse brain (KO), cerebrovascular disease (CVD), progressive supranuclear palsy (PSP), Alzheimer disease (AD), and Pick disease (PiD) brain homogenates. The fractions in the upper left corner of each panel indicate the ThT-positive/total replicate reactions

Multiple reaction conditions including temperature, shaking speed, substrate concentration, addition of salts, and co-factors were tested before arriving at the present K12 RT-QuIC reaction conditions. Using a strategy we described previously [23], we found that 400 mM NaF provided the highest fold separation of lag phases (time to threshold at which ThT fluorescence exceeded 100 standard deviations of the baseline fluorescence) between positive (AD- and PiD-seeded) and negative (KO-seeded) reactions (Additional file 2). Using these reaction conditions, we observed seeded fibrillization of the K12CFh substrate, indicated by increased ThT fluorescence, in some reactions seeded with brain homogenate dilutions as extreme as 10^− 9^ and 10^− 8^ for AD and PiD, respectively, within 50 h (Fig. 2). In contrast, brains that either lacked immunohistochemically detectable tau pathology, e.g. a cerebrovascular disease (CVD) case, or had 4R tau pathology (a PSP case), seeded fibrillization of K12CFh at only 10^− 3^ – 10^− 5^ dilution of brain homogenate. These initial experiments indicated preference of the K12CFh substrate for seeding by the tau aggregates of AD and PiD.

### K12CFh detects PiD, AD, CTE seeds preferentially over 4R tau seeds

**Fig. 3:**
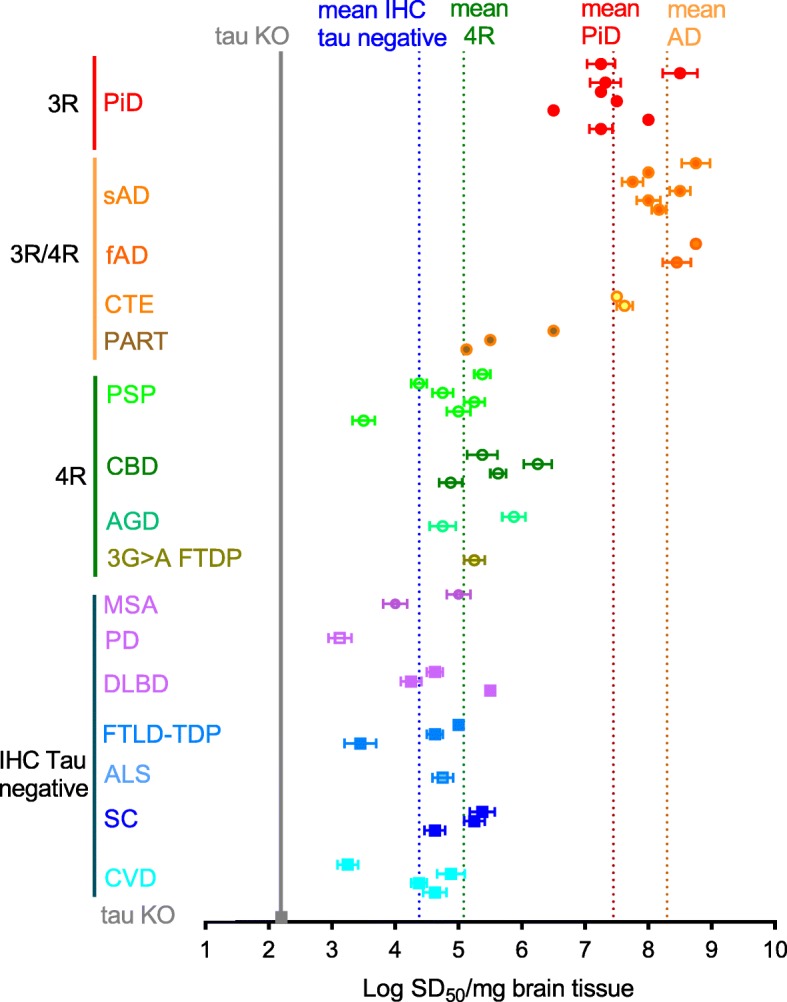
Seed concentration (LogSD_50_/mg brain) determinations of 3R, 3R/4R, 4R, and immunohistochemically (IHC) tau-negative brain homogenates by K12 RT-QuIC. Points represent logSD_50_/mg brain values calculated for a dilution series with a brain homogenate with 8 replicate reactions at each dilution. Error bars represent the standard error of logSD_50_/mg brain determined by the equation described in Methods. Vertical dotted lines represent mean logSD_50_/mg brain values across the designated brain homogenate types. PiD, Pick disease; sAD, sporadic AD; fAD, familial AD; CTE, chronic traumatic encephalopathy; PART, primary age-related tauopathy; PSP, progressive supranuclear palsy; CBD, corticobasal degeneration; AGD, argyrophilic grain disease; IVS10 + 3G > A FTDP, frontotemporal dementia with parkinsonism; MSA, multiple system atrophy; PD, Parkinson’s disease; DLBD, diffuse Lewy body dementia; FTLD-TDP43, frontotemporal lobar degeneration with TDP43 protein; ALS, amyotrophic lateral sclerosis; SC, senile change; CVD, cerebrovascular disease

To further explore the seeding selectivity of the K12CFh substrate, we performed serial dilution analyses on several brain homogenates with confirmed 3R pathology (PiD), mixed 3R/4R pathology (sAD, fAD, CTE, PART), and controls that had either 4R pathology (PSP, CBD, AGD) or lacked tau pathology according to immunohistochemical analysis. Figure 3 summarizes these data in terms of logSD_50_/mg tissue. An SD_50_ is a unit of seeding activity that gives a positive reaction in 50% of replicate reactions. Thus logSD_50_/mg tissue values represent the concentration of seeding units measured by the assay. PiD BHs had a mean log SD_50_/mg tissue of 7.5 ± 0.6 (±standard deviation), very similar to that obtained previously using the 3R RT-QuIC assay [27]. The AD and CTE brains with 3R/4R tau pathology had similarly high tau seeding activities of 8.3 ± 0.4 (*n* = 8) and 7.5–7.6 (*n* = 2), respectively. The brains with 4R tau pathology reactions all had much lower seeding activities (mean log SD_50_/mg tissue = 5.1 ± 0.7; *n* = 13), albeit higher than those of brains that were immunohistochemically negative for tau pathology (mean log SD_50_/mg tissue = 4.5 ± 0.7; *n* = 17). Cut-off times for log SD_50_/mg tissue determinations were set to 60 h based on observations that no further PiD-, AD-, or CTE-seeded reactions exceeded 100 standard deviations of the baseline ThT fluorescence at time points beyond 60 h. Spontaneous conversion of K12CFh was not observed in tau KO-seeded wells prior to 120 h. Spearman-Kärber based estimations of log SD_50_/mg tissue values for various tau aggregation disease cases and controls are summarized in Table 1 for comparison between K12, AD (3R/4R), 3R, and 4R tau RT-QuIC assays.

### Maximum ThT fluorescence distinguishes 3R- from 3R/4R-seeded reactions

**Fig. 4:**
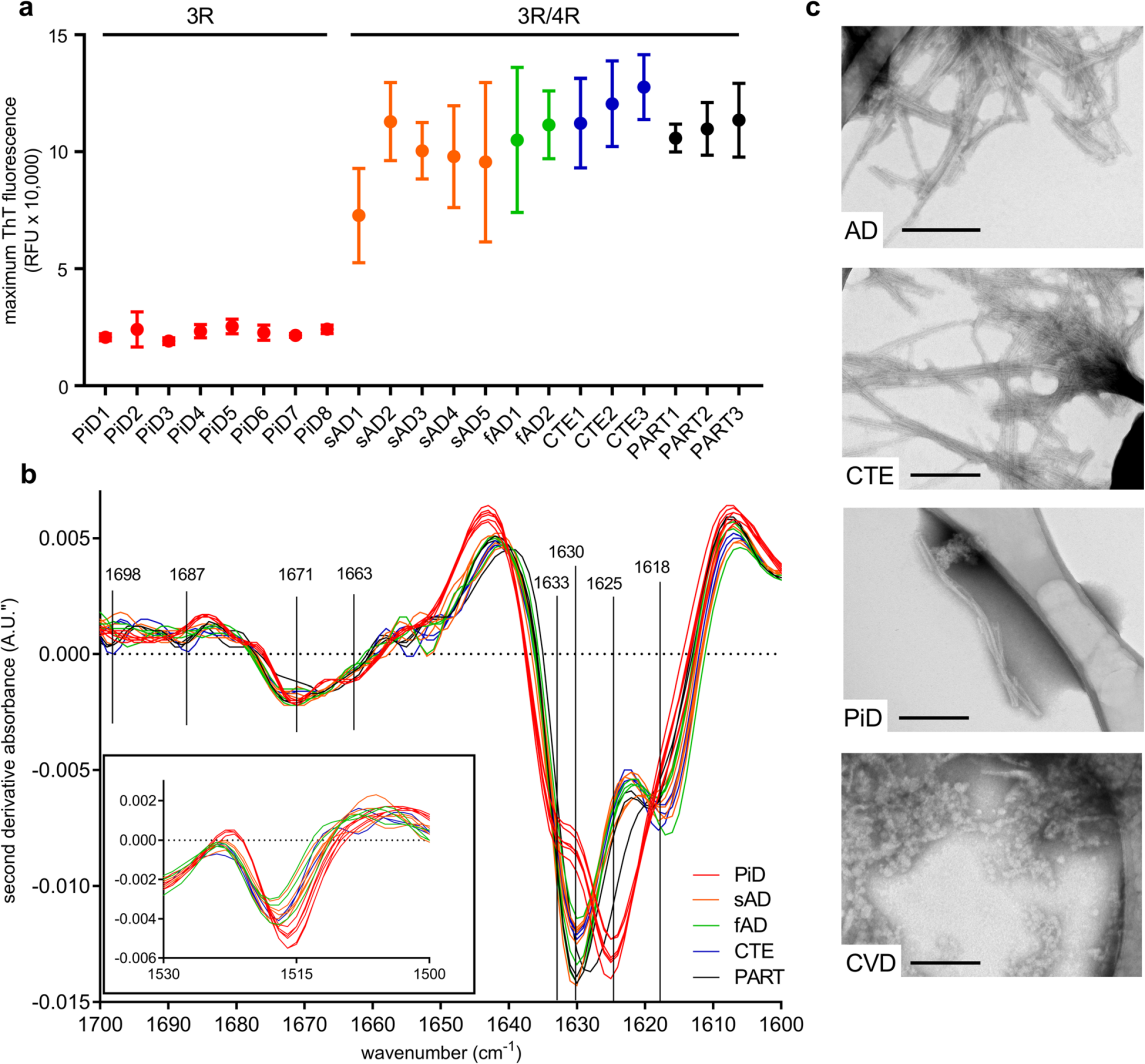
ThT amplitudes between 3R and 3R/4R-seeded K12CFh reactions correlate with FTIR banding patterns. **a**. ThT maxima within 30 h of K12 RT-QuIC reactions seeded with individual patient BHs at 10^− 4^ dilution in octuplicate; points and bars represent mean ± SD. **b**. Second derivative FTIR spectra of pooled reactions recovered from ThT maxima analysis reactions summarized in panel **a**. Each trace represents the 8 pooled reactions, seeded with a 10^− 4^ BH dilution from an individual patient. **Inset**: tyrosine vibrational mode. **c**. Transmission electron micrographs of representative 3R/4R (AD, CTE), 3R (PiD), and control (CVD) RT-QuIC products, recovered from plates at assay endpoint; bars represent 200 nm. Asterisks denote TEM grid structures

Our initial comparisons of AD- and PiD-seeded K12 RT-QuIC reactions indicated several fold higher maximum ThT fluorescence amplitudes in the AD-seeded reactions (Fig. 2, bottom two rows). Remarkably, this amplitude difference was seen over a large (10^− 3^–10^− 6^) range of BH dilutions indicating that it was primarily the character, rather than the concentration, of the seeds in the AD versus PiD brain specimens that determined the divergent fluorescence amplitudes. These fluorescence maxima differences were also seen in comparisons of reactions seeded with 8 PiD brain homogenates (~ 21,000 relative fluorescence units) compared to 2 fAD, 6 sAD, 2 CTE, and 3 PART brain homogenates (~ 50,000 - 150,000) (Fig. 4a). Modifying reaction conditions such as temperature, beads, fluorescence gain setting, or concentrations of K12CFh, ThT, NaF, and heparin all changed absolute maximum ThT values, but relative amplitude differences between PiD- and AD-seeded reactions remained (Additional file 3). These results indicated that PiD and AD seeds induced the formation of distinct K12 RT-QuIC products over a range of conditions. However, at extreme (near end-point) dilutions of PiD BH, the consistency of the relatively low ThT maxima was diminished, as observed with a 10^− 7^ dilution in Fig. 2.

### ThT fluorescence amplitudes correlate with distinct fibril structures of 3R versus 3R/4R-seeded K12 RT-QuIC reactions

To investigate if ThT fluorescence differences correlate to structural properties of the brain homogenate-seeded fibrils, we recovered RT-QuIC products from microwell plates and analyzed them by ATR-FTIR spectroscopy. Figure 4b shows second derivative FTIR spectra of 3R-seeded (red traces) and 3R/4R-seeded (green, orange, blue traces) K12 tau RT-QuIC products. All traces suggest high β-sheet content in the reaction products with several peaks in the β-sheet region (1633, 1630, 1625, 1618 cm^− 1^). The spectra of the PiD-seeded reaction products had prominent 1633, 1625 cm^− 1^ β-sheet vibrational modes, which were distinct from 3R/4R-seeded reaction products with prominent 1630 and 1618 cm^− 1^ vibrations; these results provide evidence that two distinct conformers of K12CFh aggregates resulted from the differential seeding (3R versus 3R/4R) of K12 RT-QuIC reactions. Further spectral differences were observed in the 1655–1700 cm^− 1^ region containing turn and other parallel/antiparallel β-sheet bands as well as at 1516–7 cm^− 1^ where tyrosine absorbs (Fig. 4b inset). Even further differences were evident in the fingerprint region below 1400 cm^− 1^, which is rich in side chain vibrations (Additional file 4).

We confirmed the fibrillar morphology of K12 RT-QuIC reaction products with transmission electron microscopy which indicated abundant elongated fibrils in the AD-, PiD-, and CTE-seeded, but not in control (CVD)-seeded, K12CFh products (Fig. 4c).

### Mixed AD/PiD pathology experiments

To investigate what might be observed when assaying samples with mixed AD/PiD pathology, we took AD and PiD brain homogenates with nominally comparable logSD_50_/mg tissue values, thus representing similar (within several fold) seeding doses of the tau aggregates therein, and mixed them together in ratios ranging from 10:0–0:10 AD:PiD before analyzing ThT fluorescence amplitudes in the K12 RT-QuIC. Additional file 5 summarizes the maximum ThT values observed over decreasing AD:PiD ratio at a total BH dilution of 10^− 4^. Reactions seeded only with AD BH (10:0 AD:PiD) showed characteristically high ThT fluorescence values that decrease with a decreasing ratio of AD:PiD. However, surprisingly, even with the addition of 1/10 equivalent of PiD BH, the ThT fluorescence maxima of the reactions dropped to levels approaching those obtained with PiD BH alone. These results suggested that either (1) PiD tau seeds are more efficient at seeding K12CFh fibrillization, or (2) that this PiD BH had a several fold higher seed concentration than the AD BH, which is possible because of ~ 0.5 log margin of error that we have observed is typical of end-point dilution RT-QuIC titrations.

### K12 RT-QuIC is as sensitive as AD RT-QuIC

**Fig. 5:**
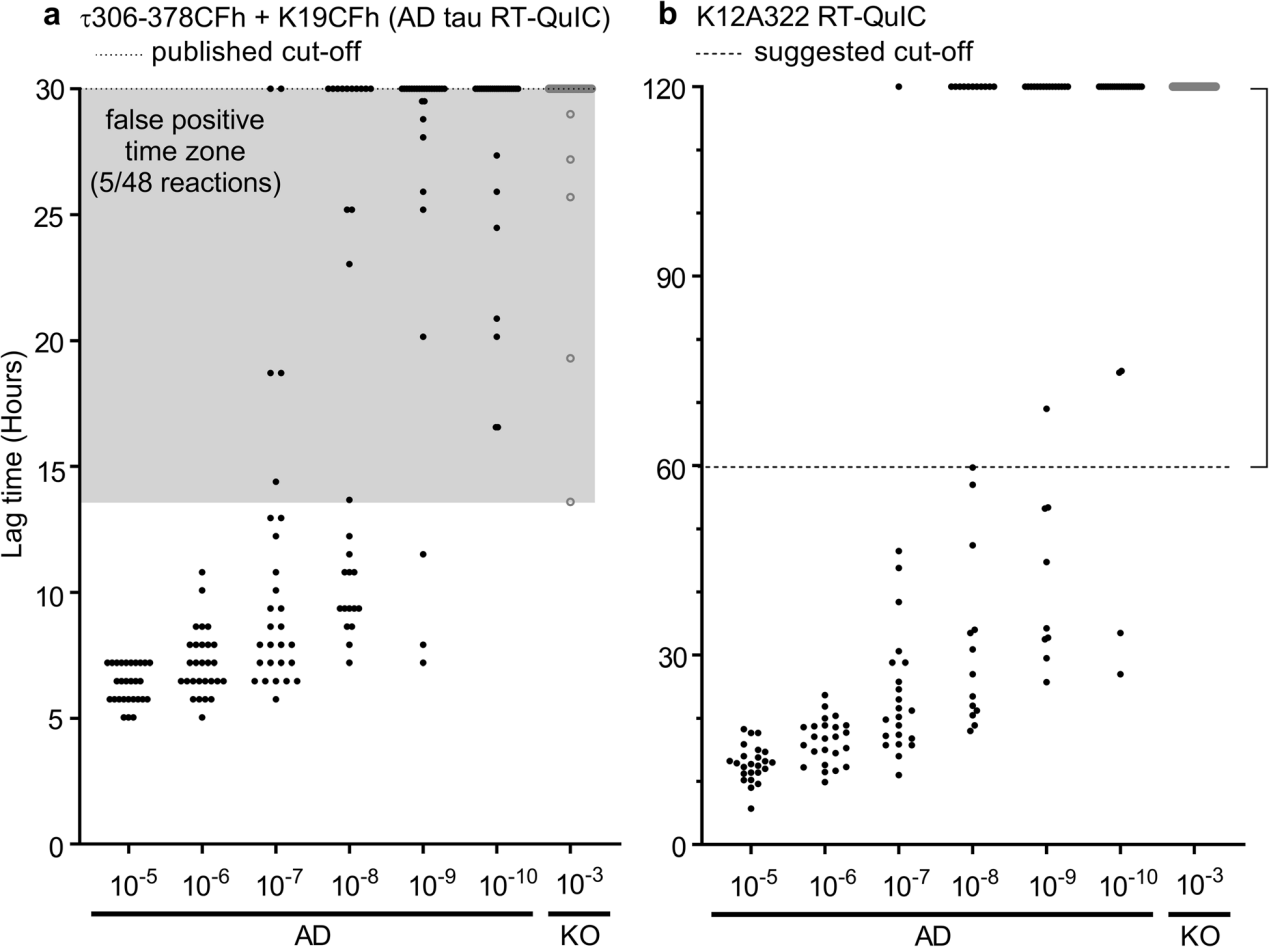
Comparison of the sensitivity of the AD RT-QuIC (**a**) and K12 RT-QuIC (**b**) assays. Data points represent the time for an individual reaction to exceed a ThT fluorescence threshold of 100 * SD of baseline fluorescence for the designated BH and dilution. Horizontally aligned overlapping points at 30 h (AD RT-QuIC) and 120 h (K12 RT-QuIC) represent reactions for which ThT fluorescence did not exceed the threshold, indicating no fibrils formed. Shaded area in **a** shows reaction times during which at least one replicate KO-seeded reaction exceeded the threshold. Bracket on upper right indicates time period in which the lack of spontaneous (KO-seeded) false positive reactions well beyond the assay cut-off time (60 h) enhances confidence in the true positives seeded by AD brain. Different lag time scales are used in panels **a** and **b** because of the different seeded polymerization kinetics obtained with the two assays. The 60–120 h timeframe is shown in **b** to document the increased stability of the K12 RT-QuIC with respect to spontaneous nucleation and polymerization in the absence of any tau seeds (i.e. in the presence of KO brain homogenate)

By performing K12 RT-QuIC analysis on serial dilutions of synthetic K12CFh fibrils, we were able to crudely estimate the concentration of fibrils that the K12 RT-QuIC assay can detect. Additional file 6 shows lag time analysis of serial dilutions of synthetic K12CFh fibrils generated by seeding with tau KO mouse BH (left), AD BH (middle), and PiD BH (right) at known fibril concentrations in K12 RT-QuIC. Individual points represent the time to threshold ThT fluorescence (fluorescence > 100*SD baseline) for 8 replicate reactions at the indicated dilutions. By Spearman-Kärber analysis, the analytical sensitivity of the assay was roughly ~ 75 fM for synthetic AD-seeded K12CFh fibrils, and ~ 7.5 fM for both synthetic PiD-seeded K12CFh fibrils and spontaneously converted (tau KO mouse-seeded) fibrils, comparable to the analytical sensitivity of the previously published AD tau RT-QuIC [21]. Finally, we compared the AD and K12 RT-QuIC assays by endpoint analysis of 7 AD BHs. Figure 5 shows the lag time, defined above, for 7 AD BHs assayed in AD RT-QuIC (**5A**) versus in K12 RT-QuIC (**5B**). Grey circles in both panels represent KO BH-seeded reactions, which serve as negative controls. The AD RT-QuIC showed occasional (5/48) positive reactions occurring in KO-seeded wells, prior to the published assay endpoint of 30 h (grey shaded area). Nonetheless, similar mean seed concentrations for AD brain samples were obtained with Spearman-Kärber analyses of end-point dilution data the two assays, i.e., 8.7 +/− 0.2 and 8.4 +/− 0.2 log SD_50_/mg with AD tau RT-QuIC and K12 RT-QuIC, respectively.

## Discussion

Given that tau deposition appears to underlie the pathogenesis of multiple neurodegenerative diseases, it is important to have robust and practical assays for different types of tau aggregates for use in diagnostics and research. Tau RT-QuIC assays exploit the fundamental self-propagating activity of pathological forms of tau, offering detection methods that are more sensitive than, and complementary to, conventional methods such as immunohistochemistry, immunoblotting, and ELISA. Thus, tau RT-QuIC assays allow measurements of multiple types of tau aggregates whether they represent the predominant cause of neurodegeneration or much less abundant co-pathology that is secondary to another disease process. Our K12 RT-QuIC assay allows the ultrasensitive detection and discrimination of both 3R and 3R/4R types of pathological tau using a single tau substrate (K12CFh). From a practical perspective, this assay halves the number of assays and recombinant tau substrates required to measure these types of tau seeds. More fundamentally, our findings also demonstrate the different templating activities of 3R versus 3R/4R tau-associated aggregates. However, our observation that the discriminatory ability of the assay based on maximum ThT fluorescence can break down with extreme near-end-point dilutions of BH (Fig. 2) suggests that 3R vs 3R/4R discrimination should be performed on specimens with seed concentrations that are at least 10-fold above the end-point dilution of the assay. Based on our current observations, K12 RT-QuIC could be applied to *postmortem* brain samples to aid in the characterization of decedents’ molecular pathologies for diagnostic and research purposes. Presumably, it can be similarly applied in animal models in pathogenesis or preclinical therapeutic studies to follow the accumulation of 3R and 3R/4R tau aggregates. Applications to *antemortem* diagnosis or therapeutic trials in humans will depend largely upon the ability to detect tau seeds in accessible tissue or fluid specimens such as CSF, but such capability remains to be shown.

A key to success in developing RT-QuIC or other seed amplification assays is maximizing the kinetic difference between seeded and unseeded (spontaneous) polymerization of the substrate molecules. In our experience the K12CFh substrate is readily seeded but is less prone to spontaneous polymerization than the tau fragments that we have used in our previous tau RT-QuIC assays [21, 23, 28, 29]. This feature can improve assay sensitivity and specificity, which may be crucial for detecting aggregates in accessible diagnostic specimens with relatively low tau seed concentrations. Recently we have shown for six different RT-QuIC assays that modification of the main anionic component in the reaction mixture can also help to delay spontaneous fibril formation [23]. In the case of the AD tau RT-QuIC assay, this strategy made it possible to conduct the assay using only the τ306–378 fragment in the presence of weakly-hydrated anions (NaBr, NaI, NaClO_4_). Thus, overall, we have simplified the amplification of AD tau aggregates by two methods: (1) by modifying the ionic species present in the reaction mixture of the original AD tau RT-QuIC [23] and (2) by extending the τ306–378 fragment N-terminally to include R1, and C-terminally to residue 400 in this work.

The ability of K12 RT-QuIC to distinguish PiD- from 3R/4R-seeded reactions based on the ThT fluorescence maxima and FTIR spectra of the reaction products suggests that at least some of the distinct conformational features of PiD and 3R/4R tau filaments can be imposed on, and propagated by, the K12CFh tau fragment. The 1618–1633 cm^− 1^ range of the major spectral differences indicated that they likely reflect variations in β-sheets formed by the polypeptide backbone because, although side-chain vibrations can absorb in this range, they typically make up only about 10% of the spectral intensity [22]. Nonetheless, the spectral differences in the more sidechain-rich range below 1400 cm^− 1^ are also consistent with conformational differences. The divergent ThT maxima exhibited by the 3R- versus 3R/4R-seeded K12CFh fibrils might be due to distinct alignments of the ThT fluorophores on fibrils with different amyloid cores and/or to the differential bundling of the fibrils.

Our inability to discriminate between the 3R/4R seed subtypes (e.g. AD, CTE and PART) could be because the filament core conformations of AD and CTE, at least, are closely similar to one another [15, 16]. Moreover, PART pathology may be on a continuum with the tau pathology of AD, as both involve deposition of 3R/4R tau isoforms in the entorhinal cortex and hippocampus in neurofibrillary tangles (NFTs); however PART by definition lacks amyloid-β pathology (NFT+/Aβ−) [10, 12]. PART is further defined as having NFT pathology at or below Braak stage IV [12]. Thus, there may be only a quantitative difference between PART and AD tau deposits. This would mean that the low level of seeding activity of PART brains (mean logSD_50_/mg tissue = 4–6) reflects lesser deposition of AD-like tau. Interestingly, 2 of 3 PART samples had seeding levels comparable to that of 4R tau and IHC tau-negative brain homogenates. This intermediate seeding activity was also observed with the original AD tau RT-QuIC assay (logSD_50_/mg tissue: KO < PiD and 4R tauopathies < AD, Table 1) [21], but not in the 3R or 4R tau RT-QuIC assays (compare control logSD_50_/mg tissue values in 3R, 4R tau RT-QuIC columns with KO averages in Table 1) [27, 29]. This seeding activity could therefore be a result of PART-related tau, which has been described elsewhere as nearly universally present at some level in aged brains [6]. NFTs can even be found in the second and third decades of life [7]. Thus, as we have now observed in two assays designed to amplify AD tau filaments (here and [21]), an elevated baseline level of tau seeding activity in control brain specimens may be readily detectable due to similarities of AD and PART tau deposits and the frequent presence of the latter in the brains of clinically-normal individuals at quantities that may not be detectable by immunohistochemistry.

Pick bodies are rarely seen in AD brains. However, in PiD brains, whereas 3R tau filaments predominate, tau filaments that contain 4R tau isoforms have also been observed [37]. The fact that K12 RT-QuIC detects both PiD- and AD-associated tau seeds raises the issue of how the assay might respond quantitatively to brain samples with mixed tau pathologies. Initially, using K12 RT-QuIC, we estimated an average of 8.3 log SD_50_/mg AD brain compared to 7.45 log SD_50_/mg PiD brain (Fig. 3), which suggested a greater deposition of tau seeds in AD brains. This conclusion is difficult to disentangle, however, from the possibility of different seeding efficiencies of structurally distinct tau aggregates [14–16]. For example, our assays of AD- and PiD-seeded synthetic fibrils of predetermined concentration revealed an additional log of sensitivity in detecting the PiD-seeded fibrils (7.5 fM vs 75 fM, Additional file 6). This suggests that the templating of K12CFh onto PiD-seeded fibrils is more efficient. One possible explanation is that the more tightly bundled AD fibrils may present fewer exposed seeding surfaces for K12CFh incorporation, relative to the loosely dispersed filaments of Pick bodies [31]. Also differences in the bundling or organization of AD- versus PiD-seeded K12CFh fibrils may impede fragmentation or lateral secondary nucleation to generate new seeding surfaces during RT-QuIC reactions. When we assayed ratios of AD:PiD BHs that each had ~ 8 log SD_50_/mg (PiD 7, AD 2 in Fig. 3) we expected to see a linear reduction in ThT amplitude as the AD:PiD ratio decreased. Instead, we saw disproportionate drop in ThT fluorescence maximum even a 9:1 AD:PiD ratio (Additional file 5), suggesting a favorability of the K12CFh tau construct to adopt the PiD fold. However, it also remains possible, considering the inherent inaccuracy in end-point dilution RT-QuIC assays of several fold, that the PiD BH had a higher seed concentration than the AD BH. Nonetheless, both the data in Additional file 5 and the additional log of sensitivity of K12 RT-QuIC for the PiD-seeded synthetic fibrils (Additional file 6) are consistent with more efficient seeding of K12CFh by PiD tau seeds. Further studies on brain tissue with varying amounts of AD (3R/4R) and PiD (mainly 3R but sometimes 4R) co-pathology will be needed to fully understand the significance of these observations.

## Conclusions

In summary, we have described a single ultrasensitive assay that detects both 3R and 3R/4R tau aggregates and discriminates the seeds of AD and CTE from those of PiD. This assay simplifies the measurement of these seeds by providing a single test that can substitute for our two previously described 3R (PiD) and 3R/4R (AD) tau RT-QuIC assays, and requires the preparation of one tau substrate, herein called K12CFh, instead of the 2 required for our previous assays. Further, this single K12CFh tau fragment showed less frequent spontaneous fibrillization compared to the substrates used in the AD RT-QuIC assay, improving the confidence with which a true positive can be declared. Importantly, the analytical sensitivity of the assay is consistent with those of the previously-published AD and PiD tau RT-QuIC assays.

Surprisingly, given the strong selectivity of our previous 3R tau RT-QuIC assay, our results also show that 3R/4R tau aggregates can seed the fibrilization of a pure 3R tau fragment that is C-terminally extended. So, from a more fundamental perspective, our biophysical characterization of the products of K12 RT-QuIC provided evidence that at least some features of the original conformations of the aggregates are propagated faithfully in these fibrillization reactions, making the assay a promising tool for further studying the misfolding process or inhibitors thereof.

## Supplementary information

**Additional file 1. **Purification of K12CFh tau. Following overnight autoinduction of K12CFh-expressing *E. coli*, tau is bound to a his column and eluted over a stepwise gradient of 23–100% buffer B (46–200 mM imidazole). Designated fractions are pooled and precipitated in acetone overnight, washed, dissolved in 8 M GdnHCl in PBS, and desalted by PD-10 size exclusion column. K12CFh fractions void of GdnHCl are pooled and stored at − 80 °C for future use.

**Additional file 2.** Salt optimization of the K12 tau RT-QuIC assay. Panels represent lag time (time to threshold of ThT > 100*SD baseline fluorescence) for reactions seeded with serial dilutions of PiD and AD brain tissue in four salts at 400 mM. Top: addition of 40 μM heparin; bottom: absence of heparin.

**Additional file 3.** Robustness of ThT amplitude differences with modified reaction conditions. Panels represent mean ThT amplitude data for three PiD and three AD brain homogenate reactions seeded at 1 × 10^− 4^ dilution in K12 tau RT-QuIC. Each panel shows a variation of a reaction condition (A, tau concentration; B, ThT concentration; C, NaF concentration; D, addition of silica beads; E, heparin concentration; F, plate reader gain, G; temperature of ThT read) beyond that of the optimized conditions listed in the figure. For gain and temperature plots, the same reaction at endpoint was scanned over increasing gain and temperature of the plate holder.

**Additional file 4.** Second derivative FTIR spectra of PiD (3R)-, AD (3R/4R)- and CTE (3R/4R)-seeded K12CFh products in fingerprint region from 800 to 1200 cm^− 1^. Each trace represents spectra from eight pooled K12 RT-QuIC reaction products seeded with an individual 1 × 10^− 4^ dilution of brain homogenate. Multiple traces represent reaction products seeded with brain homogenate from separate individuals: eight PiD (red), three sAD (orange), three fAD (green), and three CTE (blue).

**Additional file 5.** K12 RT-QuIC performance in the presence of AD and PiD tau seeds. Sixteen replicate K12 RT-QuIC reactions were performed at the given ratio of AD:PiD seeds.

**Additional file 6.** Analytical sensitivity of K12 RT-QuIC seeded with synthetic K12CFh fibrils. Panels represent lag time analysis (time where ThT fluorescence exceeded 100*SD baseline fluorescence) for eight replicate reactions seeded with the designated synthetic K12CFh fibril concentrations. Assay conditions and dilution buffer were identical to those described in methods.

## Data Availability

The datasets supporting the conclusions of this article are included within the article (and its additional file(s)).

## Abbreviations

3R: 3-repeat
4R: 4-repeat
AD: Alzheimer disease
AGD: Argyrophilic grain disease
ALS: Amyotrophic lateral sclerosis
ATR-FTIR: Attenuated total reflectance Fourier-transform infrared spectroscopy
BH: Brain homogenate
CBD: Corticobasal degeneration
CSF: Cerebrospinal fluid
CTE: Chronic traumatic encephalopathy
CVD: Cerebrovascular disease
DLBD: Diffuse Lewy body dementia
fAD: Familial Alzheimer disease
FTDP 17 *MAPT*: Frontotemporal dementias associated with *MAPT* mutations
FTLD-TDP43: Frontotemporal lobar degeneration with TDP43 protein
GdnHCl: Guanidine-HCl
IVS10 + 3G > A FTDP: Frontotemporal dementia with parkinsonism
K12CFh: K12 cysteine-free +6x His
K19CFh: K19 cysteine-free +6x His
KO: Tau knock out (tau-free)
MSA: Multiple system atrophy
NaF: Sodium fluoride
PART: Primary age-related tauopathy
PBS: Phosphate buffered saline
PD: Parkinson’s disease
PiD: Pick disease
PSP: Progressive supranuclear palsy
RFU: Relative fluorescence units
RT-QuIC: Real-time quaking induced conversion
sAD: Sporadic Alzheimer disease
SC: Senile change
TEM: Transmission electron microscopy
ThT: Thioflavin T
