# Supplementary material for: A single ultrasensitive assay for detection and discrimination of tau aggregates of Alzheimer and Pick diseases
Source: Acta Neuropathol Commun. 2020 Feb 22;8:22. doi: 10.1186/s40478-020-0887-z (PMC7036215; doi:10.1186/s40478-020-0887-z)
Supplement: Supplementary file 1 [file 40478_2020_887_MOESM1_ESM.docx]

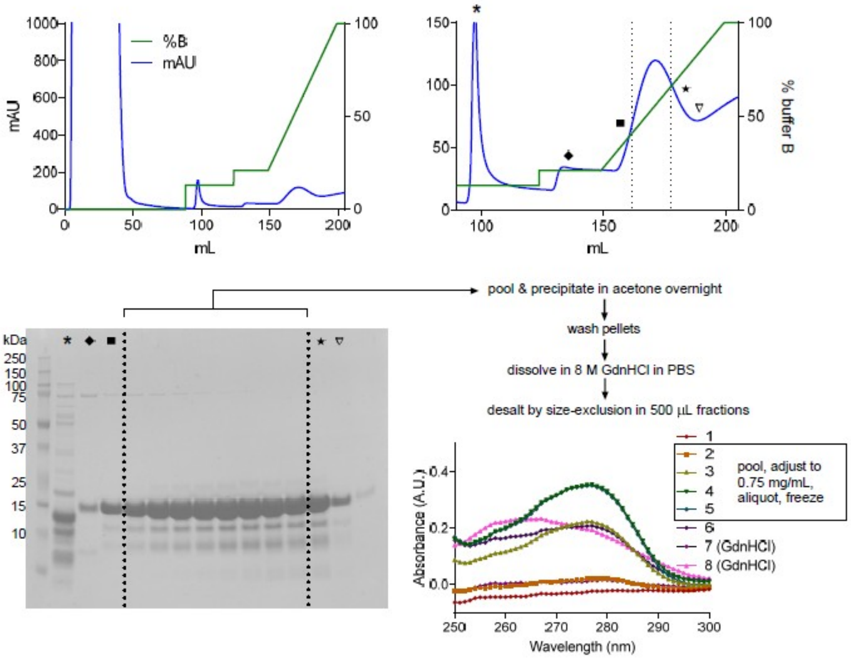

Additional file 1. Purification of K12CFh tau. Following overnight autoinduction of K12CFh expressing *E. coli*, tau is bound to a his column and eluted over a stepwise gradient of 23 – 100% buffer B (46 – 200 mM imidazole). Designated fractions are pooled and precipitated in acetone overnight, washed, dissolved in 8 M GdnHCl in PBS, and desalted by PD-10 size exclusion column. K12CFh fractions void of GdnHCl are pooled and stored at -80 °C for future use.
