# Supplementary material for: A single ultrasensitive assay for detection and discrimination of tau aggregates of Alzheimer and Pick diseases
Source: Acta Neuropathol Commun. 2020 Feb 22;8:22. doi: 10.1186/s40478-020-0887-z (PMC7036215; doi:10.1186/s40478-020-0887-z)
Supplement: Supplementary file 2 [file 40478_2020_887_MOESM2_ESM.docx]

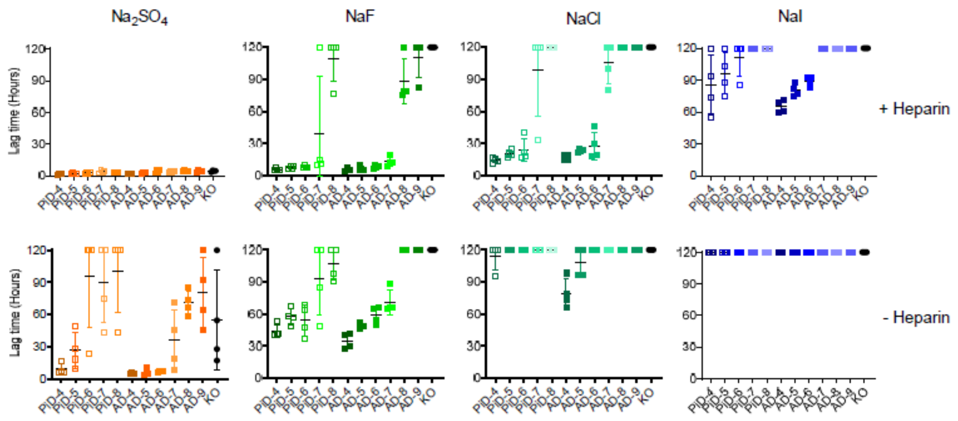

Additional file 2. Salt optimization of the K12 tau RT-QuIC assay. Panels represent lag time (time to threshold ThT > 100*SD baseline fluorescence) for reactions seeded with serial dilutions of PiD and AD brain tissue in four salts at 400 mM. Top: addition of 40 µM heparin; bottom: absence of heparin.
