# Supplementary material for: A single ultrasensitive assay for detection and discrimination of tau aggregates of Alzheimer and Pick diseases
Source: Acta Neuropathol Commun. 2020 Feb 22;8:22. doi: 10.1186/s40478-020-0887-z (PMC7036215; doi:10.1186/s40478-020-0887-z)
Supplement: Supplementary file 3 [file 40478_2020_887_MOESM3_ESM.docx]

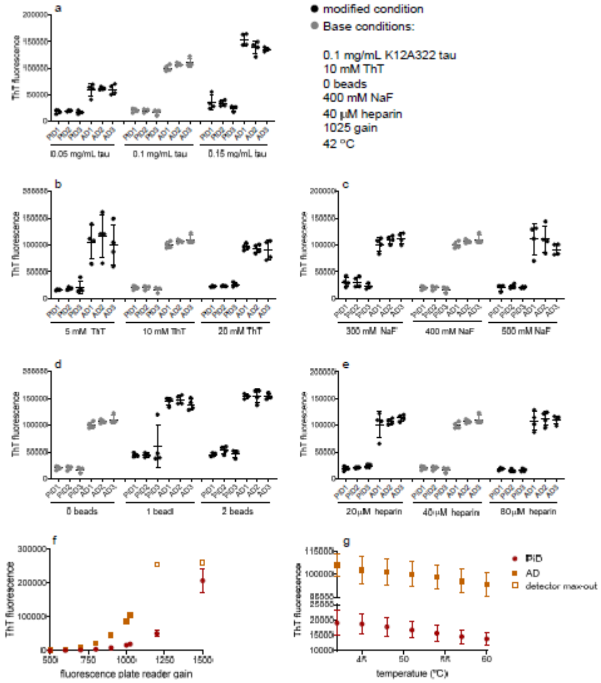

Additional file 3. Robustness of ThT amplitude differences with modified reaction conditions. Panels represent mean ThT amplitude data for three PiD and three AD brain homogenate reactions seeded at 1x10^{-4} dilution in K12 tau RT-QuIC. Each panel shows a variation of a reaction condition (A, tau concentration; B, ThT concentration; C, NaF concentration; D, addition of silica beads; E, heparin concentration; F, plate reader gain, G; temperature of ThT read) beyond that of the optimized conditions listed in the figure. For gain and temperature plots, the same reaction at endpoint was scanned over increasing gain and temperature of the plate holder.
