# Supplementary material for: A single ultrasensitive assay for detection and discrimination of tau aggregates of Alzheimer and Pick diseases
Source: Acta Neuropathol Commun. 2020 Feb 22;8:22. doi: 10.1186/s40478-020-0887-z (PMC7036215; doi:10.1186/s40478-020-0887-z)
Supplement: Supplementary file 4 [file 40478_2020_887_MOESM4_ESM.docx]

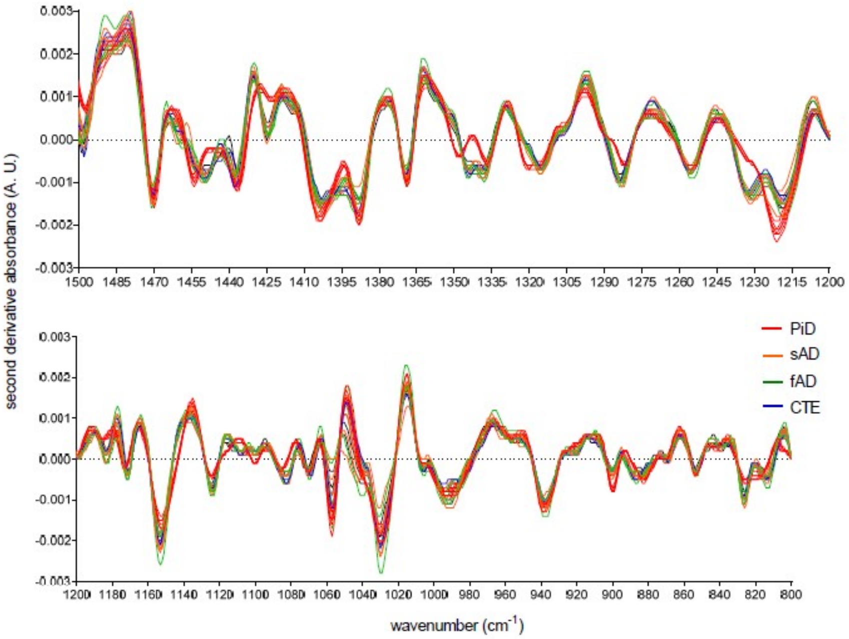

Additional file 4. Second derivative FTIR spectra of PiD (3R) and AD, CTE (3R/4R)-seeded K12CFh products in fingerprint region from 800-1200 cm^{-1}. Each trace represents spectra from eight pooled K12 RT-QuIC reaction products seeded with an individual 1x10^{-4} dilution of brain homogenate. Multiple traces represent reaction products seeded with brain homogenate from separate individuals, eight PiD (red), three sAD (orange), three fAD (green), and three CTE (blue).
