# Supplementary material for: A single ultrasensitive assay for detection and discrimination of tau aggregates of Alzheimer and Pick diseases
Source: Acta Neuropathol Commun. 2020 Feb 22;8:22. doi: 10.1186/s40478-020-0887-z (PMC7036215; doi:10.1186/s40478-020-0887-z)
Supplement: Supplementary file 5 [file 40478_2020_887_MOESM5_ESM.docx]

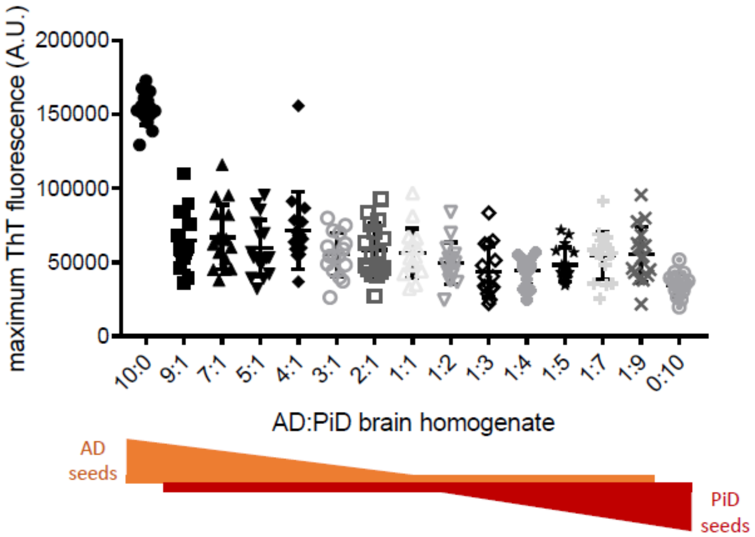

Additional file 5. K12 RT-QuIC performance in the presence of AD and PiD tau seeds. Sixteen replicate K12 RT-QuIC reactions were performed at the given ratio of AD:PiD seeds.
