# Supplementary material for: A single ultrasensitive assay for detection and discrimination of tau aggregates of Alzheimer and Pick diseases
Source: Acta Neuropathol Commun. 2020 Feb 22;8:22. doi: 10.1186/s40478-020-0887-z (PMC7036215; doi:10.1186/s40478-020-0887-z)
Supplement: Supplementary file 6 [file 40478_2020_887_MOESM6_ESM.docx]

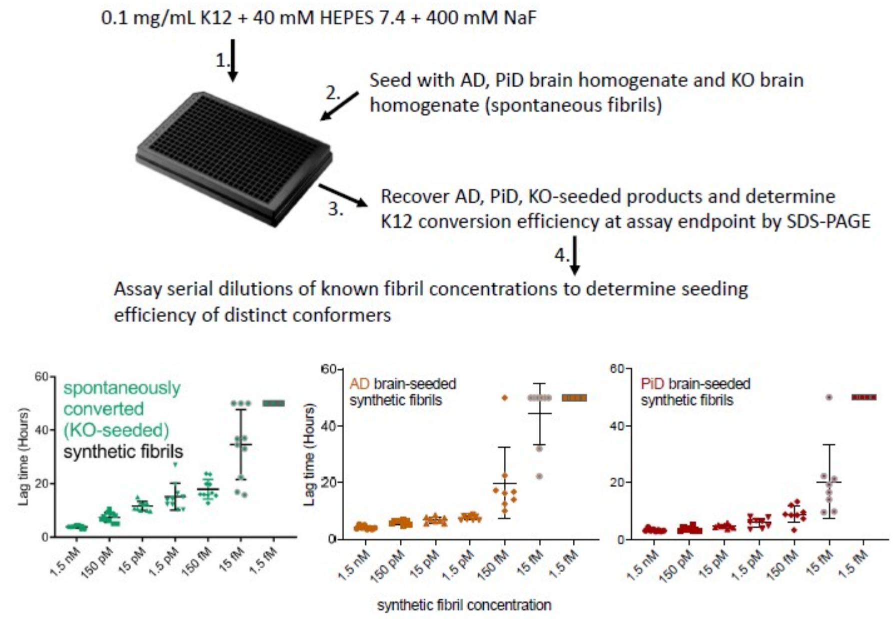

Additional file 6. Analytical sensitivity of K12 RT-QuIC seeded with synthetic K12CFh fibrils. Panels represent lag time analysis (time where ThT fluorescence exceeds 100*SD baseline fluorescence) for eight replicate reactions seeded with the designated synthetic K12CFh fibril concentrations. Assay conditions and dilution buffer are identical to those described in methods.
